# Synaptic proteins in CSF relate to Parkinson’s disease stage markers

**DOI:** 10.1038/s41531-017-0008-2

**Published:** 2017-02-08

**Authors:** Erika Bereczki, Anna Bogstedt, Kina Höglund, Panagiota Tsitsi, Lovisa Brodin, Clive Ballard, Per Svenningsson, Dag Aarsland

**Affiliations:** 1 Department of Neurobiology, Care Sciences and Society, Center for Alzheimer Research, Division for Neurogeriatrics, Karolinska Institutet, Novum, Stockholm Sweden; 2 Cardiovascular and Metabolic Diseases, Innovative Medicines and Early Development, AstraZeneca, Integrated Cardio Metabolic Centre (ICMC), Karolinska Institutet, Novum, Huddinge Sweden; 3 Institute of Neuroscience and Physiology, Department of Psychiatry and Neurochemistry Sahlgrenska Academy, Gothenburg University, 43180, Molndal, 41345 Sweden; 4 Clinical Neurochemistry Laboratory, Sahlgrenska University Hospital, Molndal, SE-431 80 Mölndal Sweden; 5 Department of Clinical Neuroscience, Karolinska Institutet, Solna, 17176 Sweden; 6 Medical School, University of Exeter, Exeter, EX1 2LU UK

## Abstract

Recent findings of morphological and functional changes in Parkinson’s disease brains have shown altered synapse formation, but their role in cognitive decline is still an area under exploration. Here we measured the concentration of three key synaptic proteins, Rab3A, SNAP25 and neurogranin by enzyme-linked immunosorbent assay, in cerebrospinal fluid from a total of 139 participants (87 controls and 52 Parkinson’s disease patients out of which 30 were drug-naïve) and explored their associations with motor and cognitive symptoms. Associations with motor disease stage (assessed by Hoehn and Yahr scale) and cognitive performance (assessed by the Montreal Cognitive Assessment scores) were explored. An overall increase in the concentration of SNAP25 was found in Parkinson’s disease patients (*p* = 0.032). Increased neurogranin levels were found in the drug naïve patients subgroup (*p* = 0.023). Significant associations were observed between increased concentration of neurogranin and cognitive impairment in total Parkinson’s disease group (*p* = 0.017), as well as in the drug naïve (*p* = 0.021) and with motor disease stage (*p* = 0.041). There were no significant disease-driven changes observed in the concentration of Rab3a. Concentrations SNAP25 and neurogranin were increased in cerebrospinal fluid of Parkinson’s disease patients in a disease specific manner and related to cognitive and motor symptom severity. Future longitudinal studies should explore whether cerebrospinal fluid synaptic proteins can predict cognitive decline in Parkinson’s disease.

## Biomarkers: Synaptic proteins give it away

The levels of synaptic proteins in the cerebrospinal fluid (CSF) correlate with Parkinson’s disease (PD) severity. Dysfunctional communication between nerve cells is an established hallmark of early stage PD and other neurodegenerative disorders. As synapses are lost from various brain areas synaptic proteins leak into the CSF, but little is known about how their concentration correlates with disease stage and prognosis. Erika Bereczki and colleagues at the Karolinska Institutet in Sweden measured the concentration of three synaptic proteins in the CSF of 52 patients with PD and 87 controls. They found that the levels of two of them (neurogranin and SNAP25) correlated with the severity of both motor and non-motor symptoms. Further work will determine whether these proteins could aid early detection of PD and help predict disease progression.

## Introduction

Parkinson’s disease (PD) is a progressive neurodegenerative disease of characteristic motor manifestations with non-motor symptoms such as depression, cognitive deficits, psychosis and sleep disturbance.^1,2^ Most patients with PD will eventually develop impairment of cognitive domains such as attention, executive and visuospatial functions and memory.^3,4^

Synaptic dysfunction is an early change in both Alzheimer's disease (AD)^5^ and PD^6^, that is, more robustly correlated with cognitive decline than neuropathological hallmarks in AD.^7,8^ Of note, *α*-synuclein and other synaptic proteins have been shown to be differentially expressed in various neurodegenerative diseases compared to non-demented controls and may have potential as biomarkers.^9–12^ *α*-synuclein seems to be deeply implicated in the synaptic vesicle trafficking as previous findings reported increased binding of *α*-synuclein aggregates to the synaptic vesicle protein Rab3A in synucleinopathies, proposing that decreased Rab3A levels are likely to directly affect not only the reserve synaptic vesicle pool but *α*-synuclein pathology as well.^13^ Accumulating evidences suggest that synaptic dysfunction is predominantly present in mutations of PARK-7, PINK1, as well as in gene mutations of LRRK2.^14–17^

We have recently reported alterations in strategic synaptic protein levels involved in key steps of the synaptic machinery in post-mortem brain studies in PD and AD.^18–21^ Neuron specific neurogranin was chosen based on its involvement in the regulation of synaptic transmission through its binding to calmodulin at low levels of calcium.^22^ SNAP25 is known to provide the driving force for vesicle fusion and docking,^23^ while the protein Rab3A reflects the recycling pool of synaptic vesicles.^24^

The objective of our current work is to further investigate the diagnostic and prognostic potential of these synaptic proteins in cerebrospinal fluid (CSF) samples from PD patients. Here we test in a proof-of principle experiment the hypotheses that CSF synaptic protein concentrations, as determined by a sensitive sandwich enzyme-linked immunosorbent assay (ELISA), are altered in PD, and associated with clinical symptoms.

## Results

### Demographical characteristics

Key cohort characteristics are shown in Table 1. There were no significant differences regarding the age or gender between total PD and control groups. As expected, PD patients receiving antiparkinson treatment had a more severe disease stage than the drug naïve, *de novo* PD patients regarding their motoric disease stage.

**Table 1:** Demographic characteristics of PD and non-neurological control group study participants

	Control	PD Total	PD Drug näive	PD Treated
N of cases	87	52	30	22
Gender M/F %	68/32	69/31	73/27	64/36
Age (years)	61.5 ± 10.2	63.9 ± 10.5	63.6 ± 11.8	64.3 ± 9
MoCA scores (44/52)	NA	23.5 ± 4.3	24.4 ± 4.1	22.4 ± 4.4
Hoehn & Yahr scale (48/52)	NA	2.0 ± 0.9	1.7 ± 0.9	2.3 ± 0.4
Disease duration(y)	NA		–	8.25 (1–18)
Medication (LED)	NA		–	790 (160–1741)

Demographic characteristics for non-neurological control and the whole as well as the stratified PD group are presented. Age, MoCA scores and Hoehn and Yahr scale is presented as mean ± SD. Disease duration is presented in years while medication is presented as L-dopa equivalent doses (LEDs). Disease duration and medication is presented as mean with lowest and highest values in parenthesis.

### Group differences of pre- and postsynaptic proteins

**Fig. 1:**
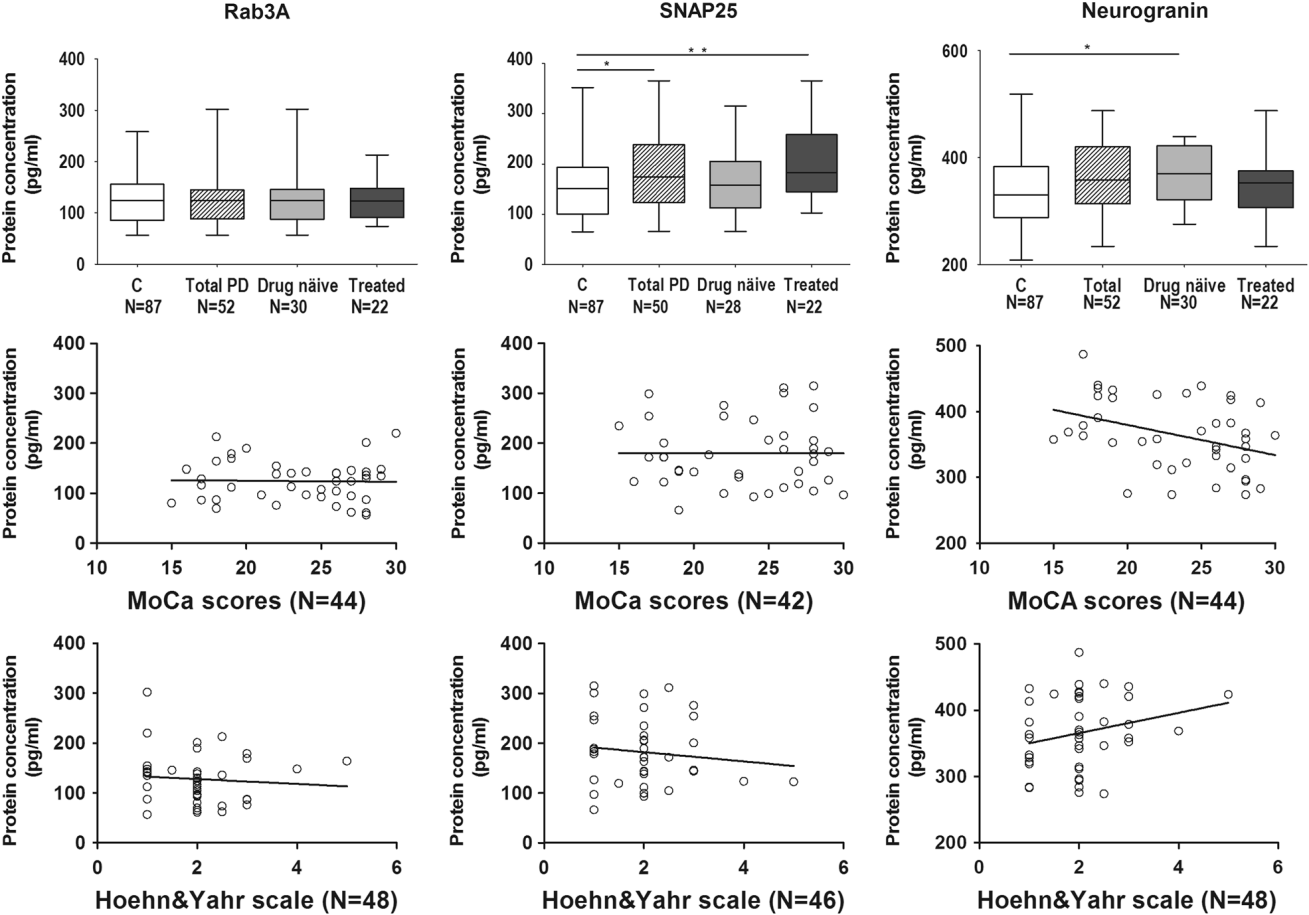
Changes in the CSF concentration of synaptic proteins neurogranin, Rab3A and SNAP25 and their clinical correlates. Concentration of synaptosome associated protein SNAP25 (**b**) measured by ELISA present overall increase in the PD patient group while presynaptic vesicle protein Rab3A (**a**) and postsynaptic protein neurogranin (**c**) concentrations remained unchanged in CSF of patients diagnosed with Parkinson disease compared to control participants. In the subgroup of drug naïve patients concentrations of neurogranin were elevated compared to control patients. Statistical analyses were performed using Mann-Whitney U test. *p*-value < 0.05 was considered significant. Neurogranin protein concentration presents significant negative correlation with cognitive impairment of PD patients assessed by MoCA scores. There were no significant correlations observed between Rab3A or SNAP25 and cognitive assessment. Neurogranin presented significant correlation with disease stage. There were no significant associations between disease stage and presynaptic proteins. The bars represent the mean values with inter-quartile range. Abbreviations used “*C* control”, “*PD* Parkinson’s disease”

The concentrations of the synaptic vesicle protein SNAP25 (*p* = 0.033) was significantly increased in the PD group 184 pg/mL compared to the control group 1600 ppg/mL (Table 2, Fig. 1b). Moreover, in the treated PD group, CSF SNAP25 concentrations were further increased to 2050 ppg/mL (*p* = 0.005). The drug naïve group (3670 ppg/mL), but not the total PD group (3590 ppg/mL), had significantly higher concentration of neurogranin levels compared to the control group (3380 ppg/mL) (*p* = 0.023) (Table 2, Fig. 1c). There were no inter-group differences in the concentration of Rab3A protein (Table 2, Fig. 1a).

**Table 2:** Differences in the synaptic proteins Rab3A, SNAP25 and neurogranin assessed by ELISA

	Control (*N* = 87)	Total PD (*N* = 52)		Drug naive PD (*N* = 30)		Treated PD (*N* = 22)	
Rab3A (pg/mL)	124 ± 4.8 *(65*–*352)*	125 ± 6.5 *(66*–*365)*	*p* = .765	124 ± 9.3 *(66*–*314)*	*p* = .831	127 ± 8.8 *(102*–*365)*	*p* = .642
SNAP25 (pg/mL)	160 ± 7.6 *(56*–*258)*	184 ± 9.9 *(57*–*302)*	*p* = .033	167 ± 12.7 *(57*–*302)*	*p* = .488	205 ± 15.1 *(74*–*213)*	*p* = .005
Neurogranin (pg/mL)	338 ± 7.4 *(208*–*518)*	359 ± 7.9 *(234*–*487)*	*p* = .057	367 ± 9.95 *(275*–*439)*	*p* = .023	347 ± 13.1 *(234*–*487)*	*p* = .571

CSF concentrations of presynaptic protein SNAP25 are increased in total PD and in treated PD patient group CSF concentration of neurogranin is significantly increased only in the drug naïve group of PD patients while CSF concentration of Rab3A remains unaffected in PD. Concentrations are expressed in pg/mL (means ± standard deviation, with minimum and maximum values in parenthesis).

*p* values represent statistical differences using Mann-Whitney U test resulting from comparisons of PD cases to non-demented control groups.

Relatively low sensitivities and specificities were found (Supplementary Table 1). Neurogranin concentration in the drug naïve group could differentiate PD patients from control with high specificity 87% but low sensitivity 37%.

### Associations between synaptic proteins with PD severity and cognitive scores

CSF synaptic protein concentrations were associated with cognitive and motor symptoms (Table 3, Fig. 1). CSF levels of neurogranin were correlated to cognitive scores assessed by MoCA (Rho = −0.358, *p* = .017, *N* = 44), i.e., higher levels associated with reduced cognition, in the total as well as the drug naïve group (Rho = −0.460, *p* = .021, *N* = 25). Increased neurogranin concentration was associated to a more advanced disease stage as assessed by the Hoehn and Yahr scale (Rho = 0.296, *p* = 0.041, *N* = 48), in particular in the early stages of disease represented by the drug naïve group (Rho = 0.473, *p* = .008, *N* = 30). In order to control for potential confounding factors such as disease duration and age, linear regression analyses were performed. Analyses before (*β* = −.355, 95% CI [−12.688, −1.261], *p* = 0.018, *R* ^2^ = 0.126) and after controlling for the impact of disease duration and age (*β* = −.345, 95% CI [−13.576, −.864], *p* = 0.027, *R* ^2^ = 0.168) revealed no substantial influence of disease duration or age related effects on correlations with cognitive scores. Similarly correlation of neurogranin with motoric disease stage remained significant after controlling for disease duration and age (*β* = −.334, 95% CI [.272, 2.411], *p* = 0.015, *R* ^2^ = 0.258).

**Table 3:** Associations of synaptic proteins with PD disease severity

	MoCA Total	Hoehn&Yahr Total	MoCA Drug naive	Hoehn&Yahr Drug naive	MoCA Treated	Hoehn&Yahr Treated
Rab3A	Rho = 0, *p* = .997, *N* = 44	Rho = −0.049, *p* = .743, *N* = 48	Rho = −0.150, *p* = .474, *N* = 25	Rho = −0.148, *p* = .436, *N* = 30	Rho = .177, *p* = .469, *N* = 19	Rho = −0.049, *p* = .743, *N* = 18
SNAP25	Rho = −0.007, *p* = .963, *N* = 42	Rho = −0.02, *p* = .897, *N* = 46	Rho = 0.115, *p* = .602, *N* = 23	Rho = −0.205, *p* = .296, *N* = 28	Rho = −0.007, *p* = .963, *N* = 19	Rho = −0.02, *p* = .897, *N* = 18
Neurogranin	Rho = −.358, *p* = .017, *N* = 44	Rho = .296, *p* = .041, *N* = 48	Rho = −.460, *p* = .021, *N* = 25	Rho = .473, *p* = .008, *N* = 30	Rho = −.491, *p* = .033, *N* = 19	Rho = .216, *p* = .390, *N* = 18

Spearman correlations are presented for Rab3A, SNAP25 and neurogranin with cognitive scores assessed by MoCA scores. Results are reported for total PD group, drug naïve group and treated patients with correlation coefficient, *p* value and number of subjects.

There were no associations between presynaptic proteins and disease severity.

## Discussion

We found that CSF concentrations of SNAP25 and neurogranin are increased in PD in a disease stage specific manner, whereas no differences were found for the synaptic vesicle protein Rab3A. The increase in the concentration of postsynaptic neurogranin was associated with reduced cognition and higher motor disease stage in PD; however their limited diagnostic and prognostic value places them on hold in the rapidly roaring biomarker field. To our knowledge, analyses of CSF SNAP25 or Rab3A from PD patients have not been previously reported.

We recently reported changes of SNAP25 and neurogranin, with little or no changes in Rab3A levels in post-mortem neocortical regions in PD and related diseases.^25^ Our CSF measurements are thus reflecting the on-going synapse loss invivo, and are in conformity with these post-mortem findings.^25^ It is widely accepted that reduced concentrations in brain of various synaptic proteins reflect the synaptic density.^26^ Increased concentrations of the very same proteins in CSF may be caused by the continuous leakage of proteins from various brain areas into the brain interstitial fluid that is cleared into the CSF.^26^ In contrast to other synaptic proteins CSF levels of *α*-synuclein are generally decreased in PD^27,28^ most likely due to the sequestration of misfolded *α*-synuclein in brain. Other studies have found that *α*-synuclein levels are increased in later stages of PD, and that increased levels are associated with cognitive decline. Thus, more studies of the *α*-synuclein levels in PD are needed. Increased levels of neurogranin, as well as SNAP25 in CSF of early prodromal AD patients have been reported^11,29^ and neurogranin levels were also correlated with cognitive impairment in prodromal AD,^10^ underlining the importance of neurogranin as a potential prognostic biomarker for neurodegenerative diseases.

Of note, disease stage and duration is likely to affect synaptic protein levels both in post-mortem brain tissue as well as in CSF. One concern often debated is whether alterations observed could be accounted for secondary drug effects. In our cohort 30 of the total 52 PD patients were de novo, drug naïve patients not receiving any antiparkinson drug at the time of CSF sampling. The CSF levels of synaptic proteins in drug naïve patients were not significantly different from the levels of treated patients although neurogranin concentrations differed between drug naïve PD and control groups. Increased neurogranin levels thus might indicate an early stage PD with marked postsynaptic loss, which is eventually equated as the disease is progressing. On the other hand docking of the synaptic vesicles, reflected by SNAP25, might be affected only later on in the disease course. This is indicated by the lack of difference between drug naïve and control patients, which became significant in more severe PD cases represented by the treated subgroup. Correlations of neurogranin with cognitive scores were significant regardless of treatment.

Recently, in a smaller cohort no overall differences were found in the levels of CSF neurogranin in PD, in contrast to increased levels in AD.^26^ Given the association with cognition in our study, it is possible that some of the synaptic proteins may be more related to amyloid pathology, which seems to be a driver of cognitive decline in PD.^30^ Whether the increase in neurogranin levels in AD, which has been previously reported,^10,12,26^ is specific only for AD or not needs to be further explored.

Limitations of the study consist in the cross-sectional design and the relatively small sample size, multiple comparisons, and the fact that not all patients completed all clinical tests. The control subjects were not tested cognitively, but history and neurological examinations did not indicate cognitive impairment or PD. Despite the small number the treated PD group is quite heterogeneous in respect of their disease duration and medication. Another limitation of our study is the lack of other relevant CSF markers such as *α*-synuclein. With these caveats in mind, our results suggest that high CSF neurogranin and SNAP25 concentrations might reflect disease stage specific synaptic impairment in PD; however future longitudinal studies should explore the predictive potential of synaptic proteins regarding cognitive decline in PD and other neurodegenerative disorders. With regards to the pathological events notably occurring early at the pre and post synaptic sites of PD patients, synaptic proteins may represent an adequate target for early therapeutic intervention.

## Materials and methods

### Participants

In total 139 subjects (87 controls and 52 PD patients) were included in the study. The participants had consented in writing to use their clinical data and CSF for research purposes, and the study was approved by the regional committee for ethics in medical research in Stockholm (PNR 2011/500-31/1 and 2012/2224-32/4). Patients were recruited at the movement disorders clinic at Karolinska University Clinic, Huddinge, Sweden. All included patients were followed longitudinally by a specialist in Movement disorders at the Department of Neurology and their diagnosis Parkinson´s disease, including subsequent responsiveness to dopaminergic therapy, was confirmed before being included in the present CSF analysis. PD patients satisfied the clinical diagnosis of PD according to the U.K. Parkinson Disease Society Brain Bank diagnostic criteria. Medications are presented as L-dopa equivalent doses (LEDs),^31^ and 30 patients were drug- naïve, de novo patients at time of lumbar puncture. PD patients underwent a clinical examination by an experienced neurologist (PS) including motor, cognitive and psychiatric symptoms. Almost all patients have available Montreal Cognitive Assessment scores (MoCA, *N* = 44)^32^ evaluating the cognitive impairment, and Hoehn & Yahr scale (*N* = 48) assessing motoric impairment. Demographic and clinical factors did not differ between PD patients with and without MoCa and Hoehn and Yahr scale scores available Control subjects were chosen in order to match in age and gender PD patients and were recruited from routine neurology examinations. Control patients were recruited at Karolinska University Clinic, Huddinge, Sweden and were diagnosed with benign neurological diagnoses, such as tension headache with no evidence of dementia, no cognitive complaints, no PD, or other brain disease.

### CSF samples

All CSF samples were obtained by lumbar puncture, collected into polypropylene-tubes and subsequently centrifuged at 1300–1800 × *g* 4 °C for 100 pmin before being stored in aliquots of 100 µl at −80 °C as previously described.^33^

### Sandwich ELISA

We have previously developed and described the sandwich ELISA for each of the studied synaptic proteins in human brain homogenates.^25^ With the exception of the synaptic-specific antibodies, the method adapted for CSF was identical regardless of the antigen. Details regarding antibodies and purified proteins are described in Supplementary Table 2. The sigmoidal standard was evaluated with non-linear four-parameter fit using SoftMax Pro 5.2 software and sample concentrations were obtained using the fitted standard curve. Each plate contained dilutions of pooled CSF as internal control and in two wells; purified standard protein was spiked in the pooled CSF in order to calculate the recovery rate. The coefficient of variation was set to be less than 20% and the accuracy of back-calculated concentration of the standard samples was set to be between 80 and 120% for acceptance. Concentrations were calculated after the mean blank value had been subtracted. The blank value was obtained from 2 wells containing all reagents except for the sample.

### Statistical analysis

As the distribution of data within the groups was not always normal, nonparametric statistics were used. Analyses (except receiver operating characteristic (ROC) curve) were performed with IBM SPSS statistics, version 22 software, while graphical illustrations were prepared in GraphPad Prism version 5. Protein concentration of controls and PD patients were compared with Mann-Whitney U test. In addition to the total PD group, drug-naïve patients were analysed separately. To assess the relationship between synaptic protein concentrations and MoCA scores, Hohn and Yahr scale, age of patients and years of disease, Spearman’s non-parametric correlations were used. Linear regression analyses were used to control for years of disease, and age. Prior to linear regression, logarithmic normalization was applied to synaptic proteins to achieve normality. In all cases differences were considered statistically significant when *p* ≤ 0.05 (*). ROC curve analysis was performed for each synaptic protein in order to assess their diagnostic value. The area under the curve, sensitivity and specificity as well as a 95% confidence interval was calculated using GraphPad Prism 5.

## Electronic supplementary material

Supplementary Information
