# Supplementary material for: Synaptic proteins in CSF relate to Parkinson’s disease stage markers
Source: NPJ Parkinsons Dis. 2017 Feb 8;3:7. doi: 10.1038/s41531-017-0008-2 (PMC5445607; doi:10.1038/s41531-017-0008-2)
Supplement: Supplementary file 1 — Supplementary Information [file 41531_2017_8_MOESM1_ESM.docx]

**Supplemental Data**

**CSF synaptic markers in PD relate to cognitive and motor symptoms**

*Erika Bereczki, PhD^1*^, Anna Bogstedt, BSc^2,3^, Kina Höglund, Phd^4,5^ , Panagiota Tsitsi MD^2^, Lovisa Brodin, MD^2^, Prof. Clive Ballard ^6^, Prof. Per Svenningsson^2^,Prof. Dag Aarsland^1^*

**^1^** Department of Neurobiology, Care Sciences and Society, Center for Alzheimer Research, Division for Neurogeriatrics, Karolinska Institutet, Novum, Stockholm, Sweden.

**^2^** Department of Clinical Neuroscience, Karolinska Institutet, 17176 Solna, Sweden.

**^3^** AstraZeneca Translational Science Centre at Karolinska Institutet, Tomtebodavagen 23a 17165 Solna, Sweden.

**^4^** Institute of Neuroscience and Physiology, Department of Psychiatry and Neurochemistry Sahlgrenska Academy, Gothenburg University, 43180 Molndal 41345.

**^5^** Clinical Neurochemistry Laboratory, Sahlgrenska University Hospital, Mölndal, SE-431 80 Mölndal, Sweden

**^6^** King’s College London, Wolfson Centre for Age-Related Diseases, London SE1 1UL, United Kingdom.

**Contents: Supplementary Table 1 and 2**

**Supplementary Table 1. Diagnostic utility of synaptic proteins**

Results from ROC curve analysis are presented with area under curve (AUC), sensitivity and specificity of synaptic proteins expressed as % with 95% confidence intervals (CI) in parenthesis. Results are reported for total groups, drug naïve group and treated patients. Models that were significant (p<0.05) are presented in bold letters.

**Supplementary Table 2. Antibodies and proteins used in the study**

Abbreviations used: Gt-goat, Rbt-rabbit, Ms-mouse, C-capture, D-detection, r-recombinant, h-human.
